# Clinical Characteristics, Treatment, and Outcomes of Peritoneal Strumosis: A Report of Three Cases and Systematic Review

**DOI:** 10.3390/diagnostics13091581

**Published:** 2023-04-28

**Authors:** Sijian Li, Xiaoxue Wang, Ruping Hong, Xinyue Zhang, Min Yin, Tianyu Zhang, Jiaxin Yang

**Affiliations:** 1 National Clinical Research Center for Obstetric and Gynecologic Diseases, Department of Obstetrics and Gynecology, Peking Union Medical College Hospital, Chinese Academy of Medical Sciences, Peking Union Medical College, Beijing 100730, China; 2 Department of Pathology, Peking Union Medical College Hospital, Chinese Academy of Medical Sciences, Peking Union Medical College, Beijing 100730, China

**Keywords:** struma ovarii, peritoneal strumosis, clinical characteristics, treatment, outcomes

## Abstract

Benign struma ovarii (SO) has a probability of metastasis named “peritoneal strumosis”, which is extremely rare, such that the specific clinical characteristics, treatment options, and survival outcomes remain unclear. We screened three cases of peritoneal strumosis among 229 cases of SO treated in our hospital. Case 1 was a 36-year-old woman with extensive peritoneal seedings at initial presentation. The second one was a 49-year-old with trocar site implant 11 years after laparoscopic adnexectomy. Case 3 was a 45-year-old woman who had an isolated lesion at the anterior surface of the rectum after laparoscopic ovarian cystectomy for SO 14 years ago. These three patients underwent surgery without any adjuvant treatment and remained disease-free after 30 to 68 months. A systematic review was then conducted and another 16 cases were identified. More than half (10/19, 52.6%) of the patients had previous SO-related ovarian surgery. The median interval between prior SO-related surgery and the initial presentation of peritoneal strumosis was 10.0 years; both regional and distant metastasis, even in the liver, lung, and heart, could also be affected. Surgery was the mainstay therapy (18/19, 94.7%), in which six patients (6/19, 31.7%) were treated with total thyroidectomy (TT) followed by radioiodine (RAI) therapy. Postoperative chemotherapy was only applied in one patient, and the last one only received a diagnostic biopsy without further treatment. Recurrence was noted in two patients with a median recurrence-free survival of 12 years, where surgical excision and RAI were then performed. No death occurred after a mean follow-up of 53 months, where 12 patients achieved no evidence of disease and five were alive with the disease. Peritoneal strumosis has unpredictable biological behaviors and the crude incidence is approximately 1.3% in SO. Patients with peritoneal strumosis have excellent survival outcomes, irrespective of different treatment strategies employed. Surgery with personalized RAI should be preferred and long-term close monitoring is recommended.

## 1. Introduction

Ovarian teratomas often contain tissue from three germ layers that typically include adipose, hair, skin, and bone tissue, accounting for approximately 20% of ovarian tumors and being the most common subtype of germ cell tumors [1]. Struma ovarii (SO) is an uncommon monodermal ovarian teratoma where thyroid tissue comprises more than 50% of all its components [2]. SO is a particularly rare subgroup that accounts for less than 5% of ovarian teratomas and less than 1% of all ovarian tumors [3]. It may present unpredictable manifestations, such as ascites, hydrothorax, elevated CA-125 level (the so-called pseudo-Meigs’ syndrome), recurrence, metastasis, and malignant transformation [4,5,6]. SO with peritoneal dissemination usually occurs in patients with malignant struma ovarii (MSO) which has been reported in about 60 cases [7]. Nonetheless, benign SO without any pathological malignant component can also present a metastasis named “peritoneal strumosis” which is extremely rare; only about ten cases are documented in the English literature [3]. Some authors may classify this situation as “highly differentiated follicular carcinoma arising in struma ovarii” for malignant biological behaviors [8]. Conservative monitoring after diagnostic biopsy, surgical excision, and aggressive treatment with the combination of surgical debulking of all the possible lesions and total thyroidectomy (TT) followed by radioiodine (RAI) therapy have been proposed in treating patients with peritoneal strumosis [6,9,10,11]. However, this data is limited and related experience only originates from case reports. Currently, there has been no treatment consensus on peritoneal strumosis due to its rarity.

To better understand and optimize the management of peritoneal strumosis, a report of three patients with this disease and a comprehensive review focused on the clinical characteristics, treatment, and survival outcomes in this special population were conducted.

## 2. Materials and Methods

The Ethics Committee of the Peking Union Medical College Hospital approved this study. First, we identified three cases of peritoneal strumosis treated in our hospital. Then, a literature review was conducted to select eligible full-length reports in the English language published between 1970 and 2022. The keywords used for searching in PubMed, Embase, and Web of Science were as follows: ‘‘metastatic struma ovarii”, “metastatic benign struma ovarii”, ‘‘peritoneal strumosis”, “highly differentiated follicular carcinoma of ovary origin”, “struma ovarii with peritoneal dissemination”, and “struma peritonei”. Relevant references cited within these articles were also reviewed. Patients with any pathologically confirmed thyroid cancer component in peritoneal strumosis, cases reported by letters or personal opinions, and non-English literature were excluded. Any imaging, pathological studies, or reports with insufficient data on clinical characteristics were also rejected. All the eligible studies were enrolled for final analysis. SO-related surgical history was defined as the surgical excision of SO or ovarian teratoma containing thyroid components. We included 16 cases of peritoneal strumosis reported in 14 studies following the screening (For details, see Supplementary Figure S1). The patients treated in our hospital were also incorporated into the overall analysis. Finally, we established a database of 19 patients, including their demographic and clinical characteristics, treatment strategies, and survival outcomes.

## 3. Results

### 3.1. Case Presentation

A total of 229 patients with SO were included for screening and only three patients (1.31%) were diagnosed with peritoneal strumosis.

#### 3.1.1. Case 1

**Figure 1:**
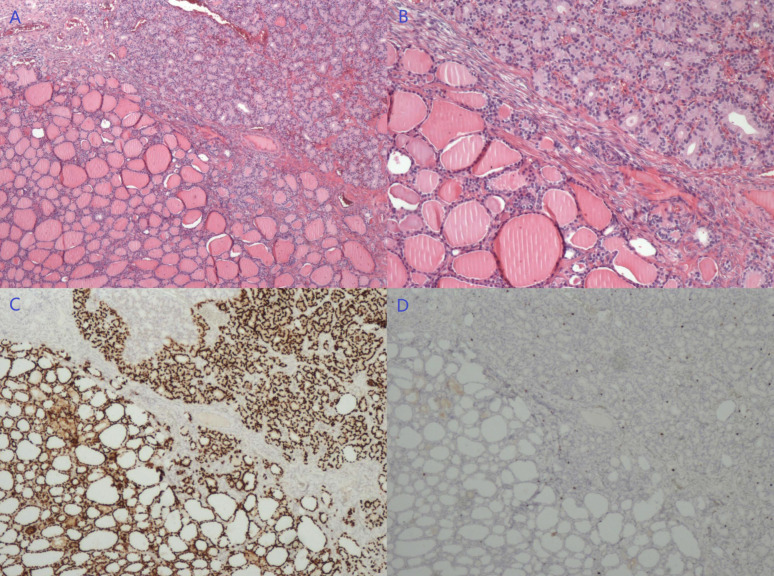
The pathology showed SO structure in the right ovarian mass (**A**,**B**, HE staining, 40×/100×), with a positive expression of TTF-1 (**C**, 40×) and Ki-67 index of 2% (**D**, 40×).

A 36-year-old female underwent laparoscopic right salpingo-oophorectomy and excision of mesentery mass due to multiple abdominal-pelvic lesions in July 2017. She denied any clinical symptoms, such as pelvic pain or hyperthyroidism. Preoperative CT revealed multiple masses of bilateral adnexa and abdominal-pelvic cavity, suggesting a right ovarian tumor with multiple peritoneal seedings; malignancy could not be excluded. However, the pathology showed SO of the right ovarian tumor and metastasis to the mesentery. An institutional pathological consultation supported this diagnosis. Immunohistochemical (IHC) staining showed positive expression of TTF-1 and the Ki-67 index was 2% (Figure 1).

**Figure 2:**
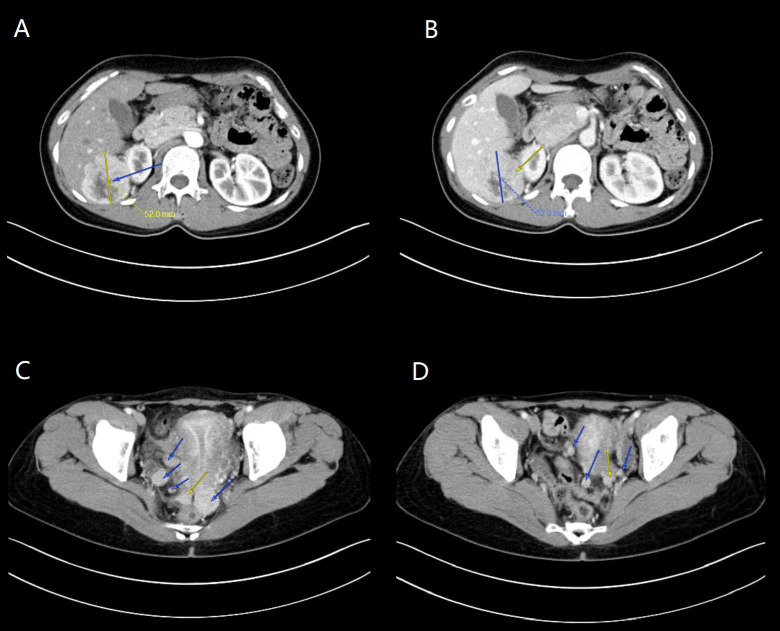
The enhanced CT examination revealed the multiple metastatic lesions in abdominopelvic cavity. (**A**,**B**, arrow) the largest tumor in right hepatorenal recess; (**C**,**D**, arrow) multiple seedings of tumors in pelvic cavity.

Repeated enhanced CT was conducted when she was referred to our hospital one month after surgery and showed a comparable result. The largest residual tumor was a solid cystic mass of 52 × 53 mm located at the right hepatorenal recess (Figure 2). Serum tumor markers, including TG, CA-125, CA19-9, CEA, AFP, and HE-4 were all negative. Thyroid function tests and ultrasound were negative. No suspicious history of ovarian teratomas was reported, and she only experienced laparoscopic left ovarian cystectomy for endometrioma 12 years ago. An exploratory laparotomy was performed to debulk all the metastases in December 2017. The intraoperative view demonstrated multiple hard nodules, ranging from 0.5 to 3 cm, seeding on the right broad ligament, posterior uterine wall, pouch of Douglas, right infundibulopelvic ligament, and the surface of the sigmoid colon. The largest tumor was a smooth, round shape at the right hepatorenal recess (Figure S2). Complete, intact excision of the previously mentioned seedings was conducted.

**Figure 3:**
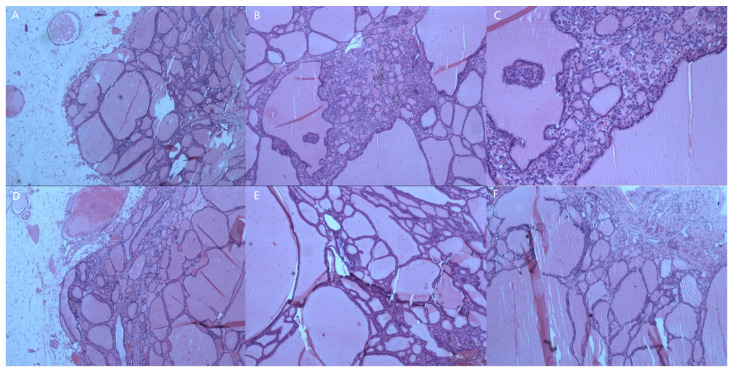
The HE staining confirmed the diagnosis of peritoneal strumosis that extensively involved the omentum (**A**, 40×), the right hepatorenal recess (**B**, 40×; **C**, 100×), the surface of sigmoid colon (**D**, 40×), right broad ligament (**E**, 40×), and posterior wall (**F**, 40×).

She recovered well and was discharged five days postoperatively without complication. The final pathology confirmed the diagnosis of peritoneal strumosis (Figure 3). Given that there was no evidence of malignancy of the tumor, she received no adjuvant therapy, and no relapse was noted at a 58-month follow-up.

#### 3.1.2. Case 2

A 49-year-old woman was referred to our hospital in February 2022 due to endometrial intraepithelial neoplasia (EIN). The only remarkable history was that she underwent laparoscopic right salpingo-oophorectomy for a teratoma in 2009.

**Figure 4:**
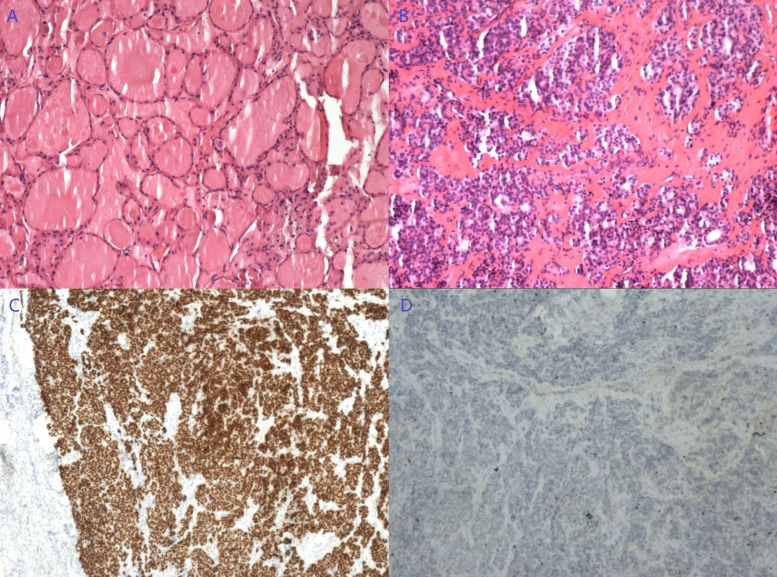
(**A**) A review of the prior surgical pathology confirmed a diagnosis of SO (HE staining, 100×). (**B**) Thyroid-like follicle structure was noted in the peritoneal nodule without evidence of malignancy (HE staining, 100×). (**C**,**D**, 40×) IHC staining showed a positive expression of TTF-1 (**C**) and Ki-67 index of 1% (**D**).

A total laparoscopic hysterectomy and left salpingo-oophorectomy were performed. Interestingly, a 2 cm smooth-round pink nodule was also noted at the right anterior peritoneum (Figure S3), while no other nodule could be found in the rest of the abdominal and pelvic cavity even after careful exploration. This nodule was completely resected intact. Pathology confirmed the EIN with a negative finding in the left adnexa. However, thyroid-like follicle structures were noted in the peritoneal nodule without evidence of malignancy (Figure 4B). IHC staining showed positive expression of TTF-1 (+), with a Ki-67 index of 1% (Figure 4C,D). A review of the prior surgical pathology was performed, resulting in a diagnosis of SO (Figure 4A). A final diagnosis of peritoneal strumosis was established based on this finding. Subsequent thyroid ultrasound and thyroid function test were both normal.

She recovered uneventfully and no further adjuvant therapy was administrated. After a regular follow-up of more than 30 months, she remains disease-free.

#### 3.1.3. Case 3

**Figure 5:**
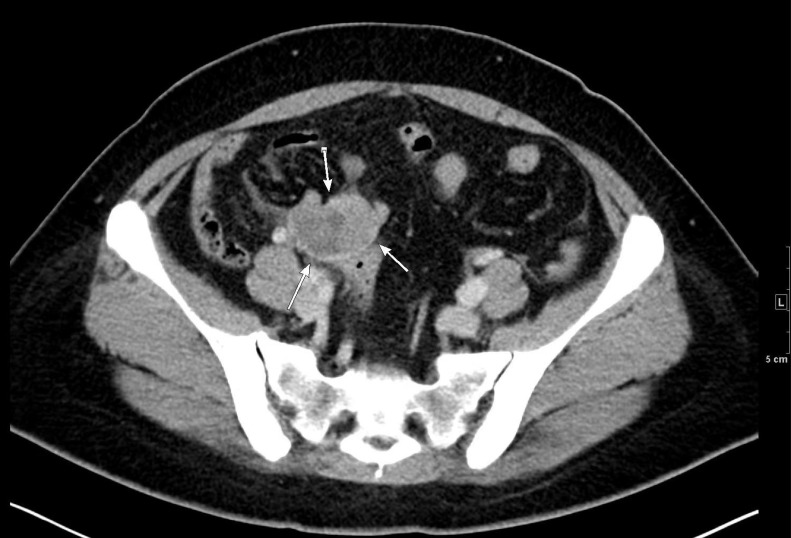
The pelvic CT revealed a 5 cm multi-locular solid-cystic mass in the pelvic cavity (the white arrows indicated the border of mass).

**Figure 6:**
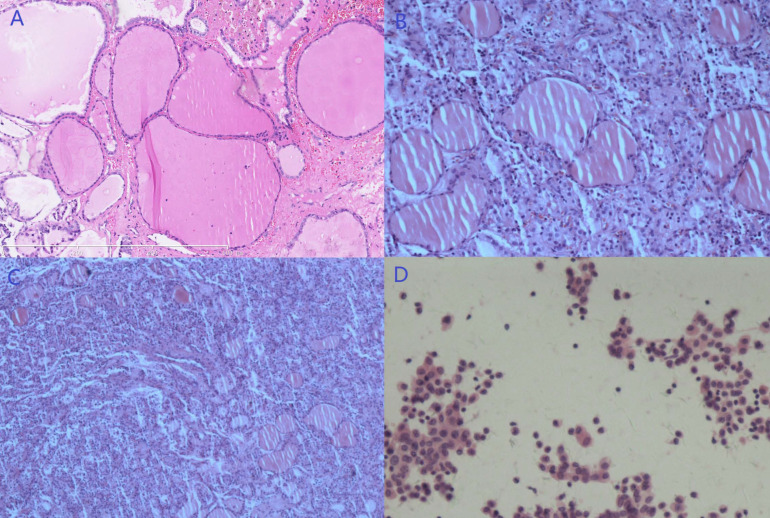
The pathology of the third patient (HE staining). (**A**) Struma ovarii arising in the right ovarian cyst (100×). (**B**,**C**) Pathology of the mesorectal mass (**B**, 100×; **C**, 40×). (**D**) No tumor cell was noted in peritoneal washings (200×).

A 45-year-old patient was admitted to our hospital due to adnexal cyst. Ultrasound and CT revealed that the multi-locular solid-cystic mass was in the right adnexal region with a size of about 5 cm (Figure 5). No obvious discomfort was complained of. The remarkable surgical history was a right ovarian cystectomy for SO 14 years ago (Figure 6A). The serum tumor markers, TG, and thyroid function were all within the normal range.

A laparoscopic exploration was performed, and a 5 cm solid-cystic mass was observed at the right anterior mesorectum with a smooth, round appearance. Neovascularization could also be noted between the tumor and mesorectum (Figure S4). No other suspicious lesion was found intraoperatively. Complete excision of the tumor was conducted, and it was removed through a specimen bag. The postoperative pathology confirmed a diagnosis of SO disseminated to the mesorectum (Figure 6B–D).

She was discharged three days after surgery and received no adjuvant therapy. No evidence of disease was noted during a 68-month follow-up.

### 3.2. Literature Review

The mean age of patients first presenting with peritoneal strumosis was 48.5 ± 11.1 years (range: 32–71). Most of them presented with an abdominal mass or nonspecific discomfort without distinct clinical manifestations. Moreover, the symptoms may relate to tumor compression, such as back pain because of vertebral involvement or vaginal bleeding caused by coexisting diseases such as endometrial intraepithelial neoplasia. Only one patient presented with thyrotoxicosis (Table 1). Peritoneal strumosis complicating a pregnancy was reported in one patient with full-term delivery through cesarean section (Table S1).

Ascites was found in two patients but their cytological pathology results were both negative. Meanwhile, peritoneal washing revealed no tumor cells noted in two other patients. Tumor markers were evaluated in 18 patients, where two presented significantly high thyroglobulin (TG, >100 ng/mL). Common tumor markers for epithelial ovarian tumors, such as CA125, were seldom positive and only two had elevated CA125. The peritoneum was the most commonly affected site (13/19, 68.4%), followed by the omentum (9/19, 47.4%), bowel (8/19, 42.1%), and uterus (5/19, 26.3%), respectively. Furthermore, the bladder, mesentery, bone, liver, spleen, heart, and lung could also be involved (Table 1 and Table S1). However, the Ki-67 proliferative index was extremely low in peritoneal strumosis where none was higher than 5% in four documented patients. SO-related surgical history was identified in 10 patients, including one with a mature teratoma containing thyroid tissue. The median intervals between SO-related surgical history and the presentation of peritoneal strumosis was 10.0 years. However, six patients underwent ovarian surgery for other pathologies, including endometrioma, simple cysts, or unspecified benign tumors. Moreover, one patient did not report any ovarian surgical history.

Surgery was the mainstay therapy in this cohort (18/19, 94.7%). One patient merely received a diagnostic biopsy without further treatment. The detailed surgical options were based on the purpose of obtaining complete excision of primary lesions and debulking all the metastatic tumors and coexisting diseases. Moreover, fertility-sparing surgery was also conducted in childbearing women. TT followed by RAI therapy was administrated in six patients at initial treatment with a total dose ranging from 60 mCi to 7.4 GBq. Synchronous thyroid cancer in the neck was found in one patient (papillary type). Postoperative chemotherapy based on adriamycin was noted in one patient. We failed to assess the impact of various surgical options and the roles of postoperative adjuvant therapies on survival outcomes because of rarity.

Follow-up information was available in 17 patients, with a median follow-up time of 32 months (range: 6–196 months). During the follow-up, two patients relapsed at 6 and 12 years later, respectively. The median recurrence-free survival was 12 years. One had tumor involving the liver, spleen, lungs, peritoneum, and bilateral adnexa regions. Biopsy and subsequent TT combined with RAI were applied with a dose of 7.4 GBq due to extensive involvement but did not achieve complete remission. The second patient obtained no evidence of disease (NED) after complete cytoreductive surgery with TT and RAI (Table S1). No death occurred at the final follow-up, 12 of them achieved NED, and the remaining five patients were alive with the disease (AWD). No potential prognostic predictor could be evaluated due to limited cases.

## 4. Discussion

Our study presents one of the largest cohorts of patients with peritoneal strumosis focusing on clinical features and survival outcomes. Patients with peritoneal strumosis mostly manifested nonspecific symptoms and usually had SO-related surgical history. Surgery was the cornerstone therapy while RAI played an important role in initial management and recurrence, especially in patients with residual or unresectable lesions. The survival outcomes were excellent regardless of therapeutic strategies, although recurrence could occur even long after treatment.

The real incidence of peritoneal strumosis has always been mysterious; no study has investigated it in previous research. Our study identified a crude incidence rate of about 1.3% among patients with SO for the first time, which accounts for less than 1/10,000 of all ovarian tumors, confirming the extreme rarity of this entity. However, peritoneal strumosis may be quiescent because its indolent nature could be neglected in patients after SO-related surgery, and the actual incidence may be underestimated. Our study demonstrated that CA125 and TG had little sensitivity in diagnosis, maybe due to the low incidence of ascites (2/19, 10.5%) and functional immaturity of the thyroid tissue components. Loizzi et al. believed that the elevation of CA125 may be secondary to the presence of ascites [12], and functional immaturity of the thyroid tissue in SO lacks the ability to synthesize TG. Furthermore, peritoneal strumosis shows no specific imaging characteristics in ultrasound, CT, and even PET/CT [13,14]. These factors make the preoperative diagnosis of peritoneal strumosis challenging, and none has successfully been correctly diagnosed before pathology.

The mechanisms of peritoneal strumosis development remain unclear. It has been found that 52.6% of the patients had SO-related surgical history; intraoperative rupture of the tumor may lead to peritoneal seedings by direct invasion, resulting in the development of peritoneal strumosis. Furthermore, the presence of lymph node involvement [11] indicates that the lymphatic metastasis pathway also existed. Furthermore, the distant metastases such as lung, bone, and heart suggested that thyroid tissue may spread through blood flow. However, the pathology of peritoneal strumosis demonstrated no infiltrating or invasion growth pattern [3]. Moreover, some patients had no history of SO-related surgical history, or the masses were removed without rupture during the operation [15]. We hypothesized that spontaneous rupture may have occurred in these patients before the presentation. Furthermore, gene mutations have been considered as potential driving factors of peritoneal strumosis in recent studies [11,16]. Brockmann et al. identified several mutations in genes ALK, EGFR, BRAF, and KRAS using next-generation sequencing (NGS). Most of these mutations were single nucleotide variants, and insertion/deletion mutations were uncommon [16]. The mutation patterns were partially similar to gene mutations in malignant struma ovarii [17], suggesting that these two diseases may share some similar pathogenesis mechanisms. Nonetheless, Bao et al. reported a patient with a germline FGFR4 Gly388Arg polymorphism who developed peritoneal strumosis from struma ovarii derived from ovarian mature teratoma. They concluded that it might contribute to the progression of peritoneal strumosis through the second hit in the developing spectrum [11]. However, most of these hypotheses originated from case reports; how the peritoneal strumosis transformed from SO still needs to be further evaluated.

Currently, the management of peritoneal strumosis remains controversial. Conservative monitoring without any treatment after diagnostic biopsy, fertility-sparing surgery with metastasectomy, and aggressive treatment combined with comprehensive cytoreductive surgery and TT followed by RAI have been reported [6,10,11,18,19]. In our study, eleven patients underwent surgery alone and six achieved NED without adjuvant therapy, including three patients treated in our hospital. This indicates that surgery alone may be sufficient in cases where complete excision of tumors is practical and being successfully conducted. However, a combination of surgery and RAI may be critical to obtaining NED in patients with extensive metastatic disease, especially in recurrent peritoneal strumosis. Moreover, RAI may be the only practical therapeutic option in unresectable peritoneal strumosis [20] for the insensitivity of chemotherapy due to its benign nature. Indeed, our study showed that most of the patients treated who received surgery and RAI had a high tumor burden; two patients experienced recurrence and were both treated with RAI. The dose of RAI mainly refers to the clinical guidelines of primary thyroid cancer, resembling malignant struma ovarii, with a reported dose ranging from 60 mCi to 7.4 GBq [5,6,21,22].

Despite the conflicting therapeutic options administered, the survival outcomes were excellent in patients with peritoneal strumosis, which reminded us to reexamine the balance of treatment and quality of life. In our cohort, no death occurred, 12 patients achieved NED, and five patients were alive with the disease, including one with a stable disease without any treatment. Although no prognostic factors were identified due to limited cases and we failed to assess the superiority of different surgical options, it is reasonable to propose that fertility-sparing surgery should be preserved in childbearing women. Conservative metastasectomy without significant impairment of physical and mental function should also be prioritized. However, the malignant biological behaviors strongly link peritoneal strumosis to preoperative suspected malignant tumors, especially epithelial ovarian cancer, which may lead to unnecessary radical surgery. Hence, evaluation including surgical history, tumor markers, imaging findings, and intraoperative frozen pathology is recommended for differential diagnoses. Moreover, our study showed a late recurrence in this population; long-term follow-up should be conducted.

This study has several limitations. The mechanisms of peritoneal strumosis development from struma ovarii are still unknown, and we did not compare the clinical characteristics in those with or without peritoneal strumosis. Furthermore, whether the malignant transformation will arise in peritoneal strumosis or not remains unclear due to the relatively short follow-up times of these reported cases. Moreover, this study only included very limited cases that could not identify any prognostic predictor of survival outcomes. Further research is warranted.

## 5. Conclusions

Peritoneal strumosis has unpredictable biological behaviors and the incidence is approximately 1.31% in SO. Patients with peritoneal strumosis have excellent survival outcomes, irrespective of different treatment strategies employed. Conservative surgery with personalized RAI may be preferred and long-term close monitoring is recommended.

## Figures and Tables

**Table 1:** The clinical characteristics of patients with peritoneal strumosis.

Items		N (Percentile)	Items	N (Percentile)
**Age (y)**		N = 19	**Sites of metastasis**	N = 19
Mean	48.5 ± 11.1		Peritoneum	13
Median	49.0 (Range 32–71)		Omentum	9
**Time of follow-up (m)**		N = 17	Bowel	8
Mean	53.0		Uterus	5
Median	32.0 (Range 6–196)		Bone	2
**Maximum mass size (cm)**		N = 17	Bladder	2
Mean	6.1 ± 3.8 (Range 2.0–15.0)		Lymph nodes	1
**Elevated tumor Makers**		N = 4	Fallopian tube	1
TG		2	Heart	1
TG + CA 125		1	Mesentery	1
CA 125		1	Right iliac fossa	1
**Synchronous primary thyroid carcinoma**		N = 19	Uterine and ovarian ligament	1
Yes		1 (5.3%)	**Recurrence**	N = 13
**SO-related surgical history**		10 (52.6%)	Yes	2 (15.4%)
**Interval to peritoneal strumosis (y)**			Mean/Median time (m)	56.8/32.0
Mean (Median)		10.5 (10.0)	**Treatment in recurrence**	N = 2
**Hyperthyroidism**		1 (5.3%)	TT + RAI	1
**Treatment options at initial presentation**		N = 19	Surgery + TT + RAI	1
Surgery alone		11	**Clinical outcomes**	N = 19
Surgery and TT + RAI		6	NED	12
Surgery and chemotherapy		1	AWD	5
No treatment (biopsy alone)		1	NA	2

Abbreviations: SO, struma ovarii; TT, total thyroidectomy; RAI, radioiodine ablation; NED, no evidence of disease; AWD, alive with disease; NA, not applicable. Notes: some patients with peritoneal strumosis involved more than one site.

## Data Availability

All data generated or analyzed during this study are included in this published article and the supplementary information files. The datasets used and/or analyzed during the current study can be obtained from the corresponding author upon reasonable request.

## Supplementary Materials

The following supporting information can be downloaded at: https://www.mdpi.com/article/10.3390/diagnostics13091581/s1, Figure S1: The detailed inclusion and exclusion process in this study (PRISMA flow diagram); Figure S2: The tumor located at the right hepatorenal recess (patient 1); Figure S3: Intraoperative findings revealed a 2 cm smooth-round pink nodule at the right anterior peritoneum (patient 2); Figure S4: Intraoperative view of the mesorectal tumor (patient 3); Table S1. The summary of patients with peritoneal strumosis [6,8,9,10,11,15,18,19,20,22,23,24,25,26].
