# Supplementary material for: Clinical Characteristics, Treatment, and Outcomes of Peritoneal Strumosis: A Report of Three Cases and Systematic Review
Source: Diagnostics (Basel). 2023 Apr 28;13(9):1581. doi: 10.3390/diagnostics13091581 (PMC10178077; doi:10.3390/diagnostics13091581)
Supplement: Supplementary file 1 [file diagnostics-13-01581-s001.zip › Table S1.pdf]

Table S1. The summary of patients with peritoneal strumosis identified from literature.

Patients	No.	Age (y)	Manifestations	Tumor size (cm)	Previous history	Metastatic sites	Treatment	Clinical outcomes
Balasch [1] et al. (1993)	1	36	persistent ovarian cyst	8*6*5	1981 (12 years previously), partial right ovariectomy (SO)	initially metastasis; multiple seedings at pelvic peritoneum, omentum and bowel, 2-3cm	1993-laparotomy BSO+metastasectomy (left SO, 8cm; R SO, 2cm)	Proposed TT and RAI, not repoted outcomes
Karseladze [2] et al. (1994)	2	49	AUB due to myoma	5*5*4	1975 (25 years previously), RSO for simple cyst	initially metastasis; multiple seedings at omentum, 0.5- 5cm	1990-laparotomy TAH+LSO+omentectomy; chemothearpy (adriamicin 60mg + farmorubicin 80mg)	NED at 32 months
Brogsitter [3] et al. (2004)	3	50	ovairan cyst	NA	none	initially metastasis, peritoneum	RSO (USO); RAI (3.7GBq) after TT	NED at 6 months
Roth et al. [4] (2008)	4	58	abdominal-pelvic mass	5	LSO for SO at 32-year-old and RSO for ectopic pregnancy at 38-year-old	para-aortic lymph node, omentum, and peritoneum	metastasectomy + appendectomy + liver biopsy; RAI after TT	AWD at 8 years
	5	49	vaginal bleeding	5	RSO for simple ovarian cyst 15 years ago	omentum	TAH+LSO	NED at 16 years and 4 months
	6	50	PMB	15	NA	omentum, peritoneum, bladder	sTAH+RSO+omentectomy+excision of the larger peritoneal nodules; TT +RAI	NED at 6 years
Kim et al. [5] (2009)	7	49	thyrotoxicosis (intermittent palpitation, facial flush); CA125 50.6U/ml, TG 179ng/ml	12*7	1996 (5 years ago) LSO for benign ovarian tumor	initially metastasis, 2cm sigmoid colon, cul-de-sac, omentum	TAH+right ovarian cystectomy+LS+metastasectomy+bilateral pelvic lymphadenectomy; TT + RAI (7.4GBq)	NED at 14 months
Akahira et al. [6] (2013)	8	64	pelvic nodules	2-3	10 years previously, mature teratoma containing thyroid tissue, rupture (RSO)	initially metastasis; pelvic cavity (uterus, rectum, and mesentery)	sampling biopsy	AWD at 17 months
Ranade et al. [7] (2015)	9	43	abdominal-pelvic mass	7*6	6 years previously, excision of SO	initially metastasis; right iliac fossa, colonic, and peritoneal nodules	metastectomy of right iliac fossa, colonic, and peritoneal nodules; recurrence at liver, spleen, lungs, multiple peritoneal and bilateral adnexal mass after 12 years, underwent biopsy, TT + RAI (7.4GBq)	AWD at 147 months, uptake increased after RAI, subsequent RAI prepared
Kaleem et al. [8] (2018)	10	70	elevated alkaline phosphatase; TG 101.5ng/ml	2-3	TAH+BSO for endometriosis 31 years ago	multiple peritoneal seedings; peritoneal washing negative	laparotomy partial omentectomy, bone marrow biopsy negative; TT (PTC in the neck) + RAI (30mCi for two courses at 20-month interval)	AWD (TG 1.3ng/ml) at 41 months, refuse to RAI
Riggs et al. [9] (2018)	11	41	pelvic pain; TG 62.4ng/ml	miliary	RSO for SO 9 years ago	uterus, peritoenum	modified radical hysterectomy	AWD (TG 65.5ng/ml) at 12 months
Dobi et al. [10] (2019)	12	52	abdominal bloating; CA125 996U/ml	12.5	None	uterus, colonic serosa, and omentum	TAH+BSO+omentectomy+rectosigmoid resection and anastomosis and left pelvic, common iliac lymph node dissection	NED at 12 months
Henderson et al. [11] (2019)	13	71	right facial swelling	4.1	excision of pelvic dermoid 48 years ago	heart, mandibular bone	excision of the cardiac tumor; TT + RAI (150/200mCi)	NED at 18 months
Li et al. [12] (2021)	14	46	Fatigue, anorexia,	3.9*3.7	laparoscopic bilateral	surface of right fallopian tube,	Cesarean section, intact left ovarian cystectomy, and	Not reported; gave birth 2970g

			abdominal swelling; CA125 124.7U/ml, spontaneously decrease to normal		ovarian cystectomy 10 years ago (intact without spillage; Left serous cystadenoma; R struma ovarii), no relapse after 8 years	uterus, urinary bladder, peritoneum, and sigmoid colon	excision of metastases	female neonatus
Bao et al. [13] (2022)	15	72	dyspnea	2	right ovarian cystectomy for SO 3 years ago and for mature teratoma 9 years ago	peritoneum, omentum	right ovarian cystectomy+lesions resection; recurrence at peritoneum, para-aortic lymph nodes, omentum, and mesorectum after 6 years, underwent LH+BSO, omentectomy, pelvic and para-aortic lymhadenectomy, excision of lesions, TT + RAI	NED at 90 months
Asaturova et al. [14] (2022)	16	55	PMB, abdominal pain	6.2*3.4*2.7 (L5 vertebra); left ovarian mass, 3.5*3.4	left ovarian cystectomy (SO) 4 years ago	vertebra (L5); omentum, surface of sigmoid colon	vertebroplasty; laparoscopic LSO (USO), omentectomy, peritoneal biopsy, and excision of sigmoid serosal lesions	NED at 17 months; fine neddle biopsy of thyroid negative

Abbreviations: N, No; NA, not applicable; TG, thyroglobulin; TM, tumor markers; SO, struma ovarii; B (R/L) SO, bilateral (right/left) salpingo-oophorectomy; TAH, total abdominal hysterectomy; LH, laparoscopic hysterectomy; TT, total thyroidectomy; RAI, radioiodine therapy; PTC, papillary thyroid cancer; NED, no evidence of disease; AWD, alive with disease.

References:

1. Balasch J, Pahisa J, Márquez M, Ordi J, Fábregues F, Puerto B, et al. Metastatic ovarian strumosis in an in-vitro fertilization patient. Hum Reprod. 1993;8(12):2075-2077.
2. Karseladze AI, Kulinitch SI. Peritoneal strumosis. Pathol Res Pract. 1994;190(11):1082-1085; discussion 1086-1088.
3. Brogsitter C, Wonsak A, Würl K, Kotzerke J. Peritoneal strumosis. Eur J Nucl Med Mol Imaging. 2004;31(7):1057.
4. Roth LM, Karseladze AI. Highly differentiated follicular carcinoma arising from struma ovarii: a report of 3 cases, a review of the literature, and a reassessment of so-called peritoneal strumosis. Int J Gynecol Pathol. 2008;27(2):213-222.
5. Kim D, Cho HC, Park JW, Lee WA, Kim YM, Chung PS, et al. Struma ovarii and peritoneal strumosis with thyrotoxicosis. Thyroid. 2009;19(3):305-308.
6. Akahira J, Endo M, Chiba R, Tanoguchi K, Yamauchi J, Ishiyama S, et al. Peritoneal strumosis, 10 years after laparoscopic surgery for mature cystic teratoma of the ovary: a case report. Int Canc Conf J. 2013;2(4):251-254.
7. Ranade R, Rachh S, Basu S. Late Manifestation of Struma Peritonei and Widespread Functioning Lesions in the Setting of Struma Ovarii Simulating Highly Differentiated Follicular Carcinoma. J Nucl Med Technol. 2015;43(3):231-233.
8. Kaleem T, Peterson J, Krishna M. Peritoneal strumosis: Presentation and management with multiple radioactive iodine treatments. J Clin Transl Endocrinol. 2018;8:1-4.
9. Riggs MJ, Kluesner JK, Miller CR. Management of highly differentiated thyroid follicular carcinoma of ovarian origin with a minimally invasive approach. Gynecol Oncol Rep. 2018;24:87-89.
10. Dobi A, Kim SA, Zhang M, Iames E, Lamzabi I. Highly differentiated follicular carcinoma of ovary: Use of imprint cytology at intraoperative consultation. Diagn Cytopathol. 2020;48(4):360-363.
11. Henderson BB, Chaubey A, Roth LM, Robboy SJ, Tarasidis G, Jones JR, et al. Whole-Genome and Segmental Homozygosity Confirm Errors in Meiosis as Etiology of Struma Ovarii. Cytogenet Genome Res. 2020;160(1):2-10.
12. Li Z, Wang J, Chen Q. Struma ovarii and peritoneal strumosis during pregnancy. BMC Pregnancy Childbirth. 2021;21(1):347.
13. Bao YT, Wang C, Huang W, Yao LQ, Yuan L. A rare case of highly differentiated follicular carcinoma in ovary with FGFR4 Gly388Arg polymorphism: a case report and literature review. J Ovarian Res. 2022;15(1):71.
14. Asaturova A, Magnaeva A, Tregubova A, Kometova V, Karamurzin Y, Martynov S, et al. Malignant Clinical Course of "Proliferative" Ovarian Struma: Diagnostic Challenges and Treatment Pitfalls. Diagnostics (Basel). 2022;12(6).
