# Supplementary figures and images for: Clinical Characteristics, Treatment, and Outcomes of Peritoneal Strumosis: A Report of Three Cases and Systematic Review
Source: Diagnostics (Basel). 2023 Apr 28;13(9):1581. doi: 10.3390/diagnostics13091581 (PMC10178077; doi:10.3390/diagnostics13091581)

Figure S1. The screening details according to PRISMA of this study.

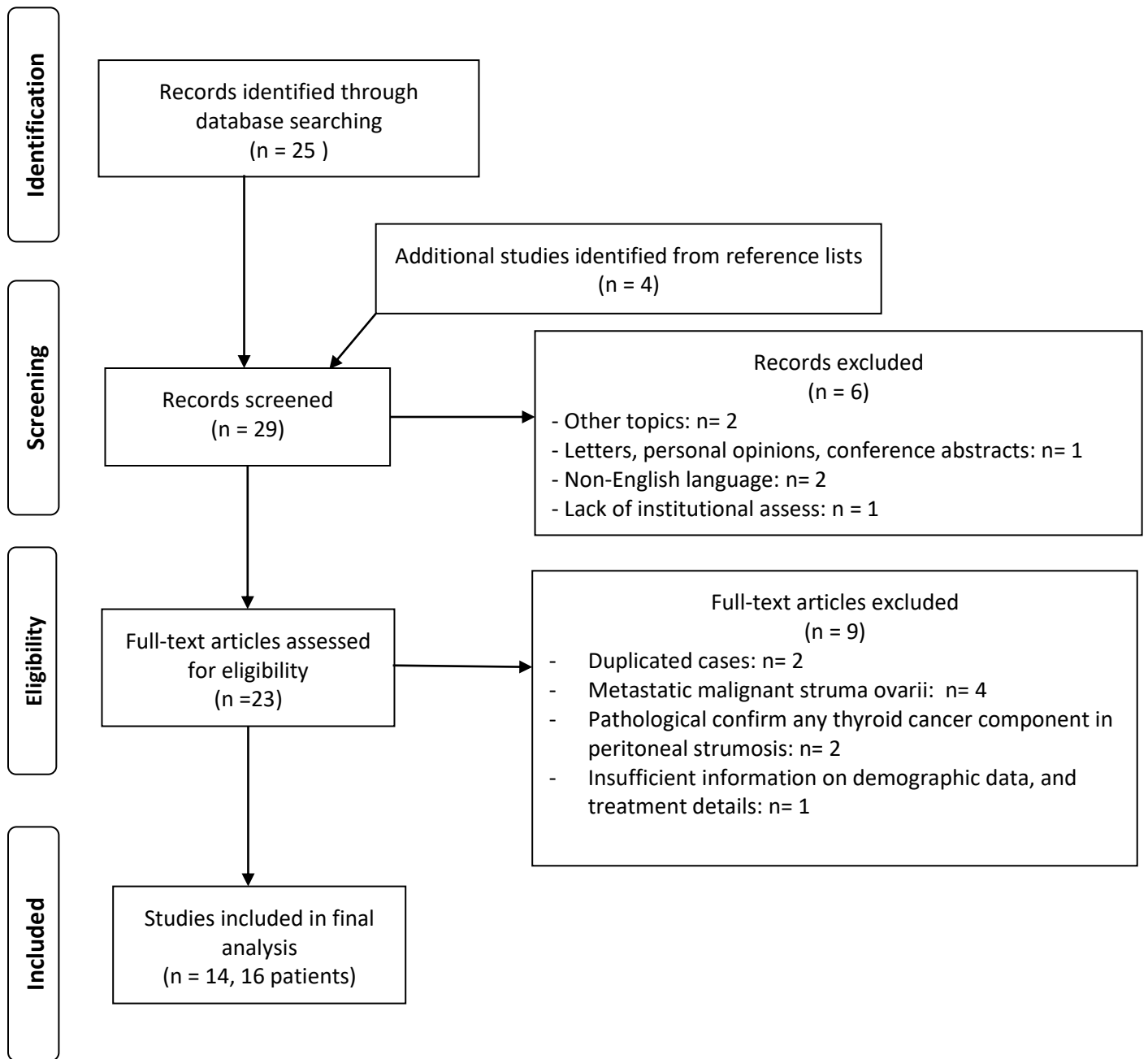

Supplement: Supplementary file 1 [file diagnostics-13-01581-s001.zip › Figure S1. PRISMA flow diagram.pdf]

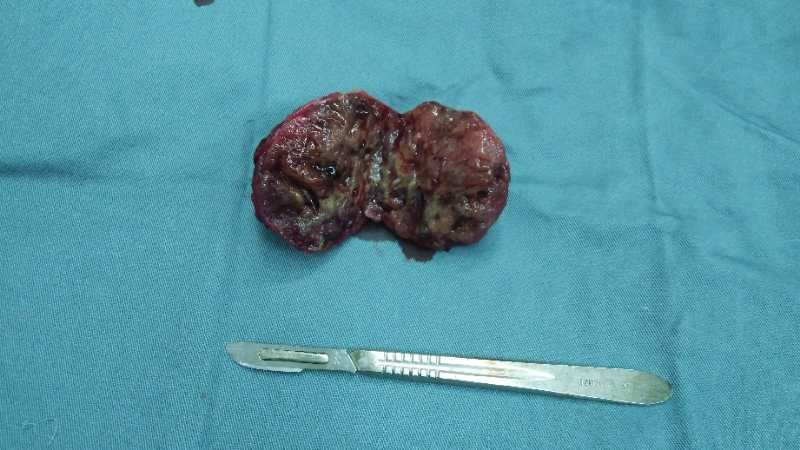

Supplement: Supplementary file 1 [file diagnostics-13-01581-s001.zip › Figure S2.jpg]

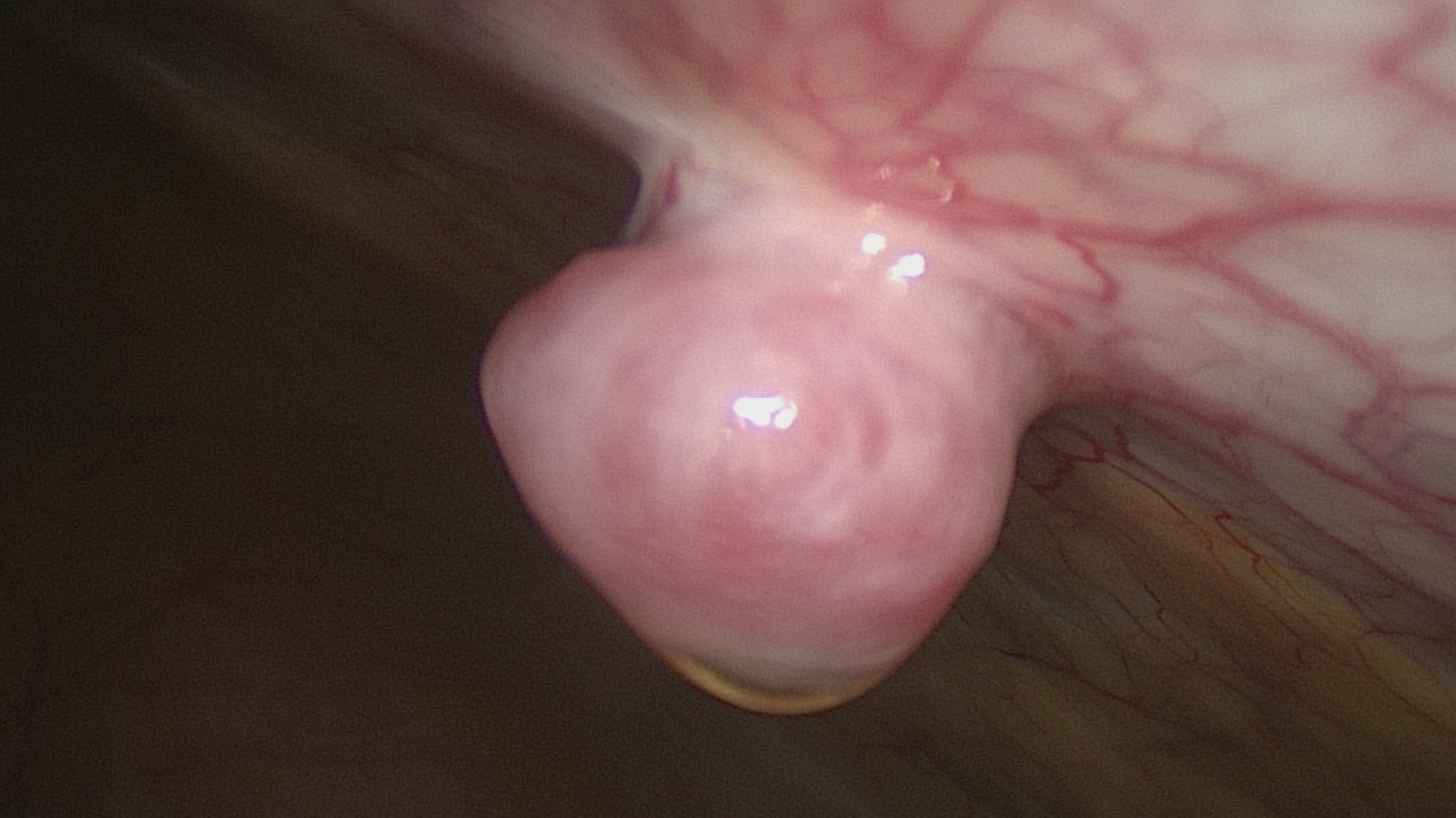

Supplement: Supplementary file 1 [file diagnostics-13-01581-s001.zip › Figure S3.jpg]

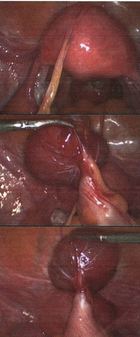

Supplement: Supplementary file 1 [file diagnostics-13-01581-s001.zip › Figure S4.JPG]
